# Recovery of strain-resolved genomes from human microbiome through an integration framework of single-cell genomics and metagenomics

**DOI:** 10.1186/s40168-021-01152-4

**Published:** 2021-10-12

**Authors:** Koji Arikawa, Keigo Ide, Masato Kogawa, Tatsuya Saeki, Takuya Yoda, Taruho Endoh, Ayumi Matsuhashi, Haruko Takeyama, Masahito Hosokawa

**Affiliations:** 1 bitBiome, Inc., 513 Wasedatsurumaki-cho, Shinjuku-ku, Tokyo, 162-0041 Japan; 2 Department of Life Science and Medical Bioscience, Waseda University, 2-2 Wakamatsu-cho, Shinjuku-ku, Tokyo, 162-8480 Japan; 3 Computational Bio Big-Data Open Innovation Laboratory, National Institute of Advanced Industrial Science and Technology, 3-4-1 Okubo, Shinjuku-ku, Tokyo, 169-8555 Japan; 4 Research Organization for Nano and Life Innovation, Waseda University, 513 Wasedatsurumaki-cho, Shinjuku-ku, Tokyo, 162-0041 Japan; 5 Institute for Advanced Research of Biosystem Dynamics, Waseda Research Institute for Science and Engineering, 3-4-1 Okubo, Shinjuku-ku, Tokyo, 169-8555 Japan

**Keywords:** Single-cell genomics, Metagenomics, Binning, Software

## Abstract

**Background:**

Obtaining high-quality (HQ) reference genomes from microbial communities is crucial for understanding the phylogeny and function of uncultured microbes in complex microbial ecosystems. Despite improvements in bioinformatic approaches to generate curated metagenome-assembled genomes (MAGs), existing metagenome binners obtain population consensus genomes but they are nowhere comparable to genomes sequenced from isolates in terms of strain level resolution. Here, we present a framework for the integration of single-cell genomics and metagenomics, referred to as single-cell (sc) metagenomics, to reconstruct strain-resolved genomes from microbial communities at once.

**Results:**

Our sc-metagenomics integration framework, termed SMAGLinker, uses single-cell amplified genomes (SAGs) generated using microfluidic technology as binning guides and integrates them with metagenome-assembled genomes (MAGs) to recover improved draft genomes. We compared sc-metagenomics with the metagenomics-alone approach using conventional metagenome binners. The sc-metagenomics approach showed precise contig binning and higher recovery rates (>97%) of rRNA and plasmids than conventional metagenomics in genome reconstruction from the cell mock community. In human microbiota samples, sc-metagenomics recovered the largest number of genomes with a total of 103 gut microbial genomes (21 HQ, with 65 showing >90% completeness) and 45 skin microbial genomes (10 HQ, with 40 showing >90% completeness), respectively. Conventional metagenomics recovered one *Staphylococcus hominis* genome, whereas sc-metagenomics recovered two *S. hominis* genomes from identical skin microbiota sample. Single-cell sequencing revealed that these *S. hominis* genomes were derived from two distinct strains harboring specifically different plasmids. We found that all conventional *S. hominis* MAGs had a substantial lack or excess of genome sequences and contamination from other *Staphylococcus* species (*S. epidermidis).*

**Conclusions:**

SMAGLinker enabled us to obtain strain-resolved genomes in the mock community and human microbiota samples by assigning metagenomic sequences correctly and covering both highly conserved genes such as rRNA genes and unique extrachromosomal elements, including plasmids. SMAGLinker will provide HQ genomes that are difficult to obtain using metagenomics alone and will facilitate the understanding of microbial ecosystems by elucidating detailed metabolic pathways and horizontal gene transfer networks. SMAGLinker is available at https://github.com/kojiari/smaglinker.

Video abstract

**Supplementary Information:**

The online version contains supplementary material available at 10.1186/s40168-021-01152-4.

## Background

The accumulation of reference genomes from microbes has provided insights into the ecology and evolution of environmental and host-associated microbiomes. The gold standard for microbial genome sequencing has been to culture specific strains and sequence extracted DNA [1–3]. Metagenomic analysis, which combines the direct extraction of genomic DNA from the microbial community with an *in silico* reconstruction of each microbial genome sequence from massive sequenced reads, has attracted much attention. A growing number of metagenome-assembled genomes (MAGs) have increased our understanding of microbial diversity in various environments [4–9].

In a metagenomic approach, genome reconstruction is performed in two steps: (1) assembling fragmented genome sequences to contigs and (2) binning contigs into lineages as bins. State-of-the-art binners rely on nucleotide compositional information such as tetranucleotide frequency, GC content, or sequence coverage [10–12]. However, these tools demonstrate different performances and produce different MAGs, including incomplete bins and multi-species composite bins [13]. Composite genomes that aggregate sequences originating from multiple distinct species or strains can yield misleading insights if they are registered as single genomes in the reference database [14]. To solve these problems, several approaches combine and curate the result of multiple binners to generate a large number of high-quality (HQ) genomes [13, 15, 16]. However, in real-world samples, it is difficult to verify binning results because there are numerous microbes without the reference genome and the proportion of microbial species richness among them is unknown.

Single-cell genomics is an alternative approach for culture-independent sequencing of microbial genomes [17]. In contrast to metagenomics, single-cell genomics does not require microbial population clonality but instead recovers genome sequences from individual cells. In single-cell genomics, DNA amplification often causes amplification biases and incompleteness in genome sequences. Therefore, co-assembly of individual single-cell sequencing data is generally required to compensate for the gaps and errors in each single-cell amplified genome (SAG) sequence [18]. However, most SAGs generally have low completeness, and even with co-assembly, produce shortly fragmented contigs, rarely covering the entire genome.

Metagenomics assesses the genomes of all microbes present in a sample, whereas single-cell genomics reveals individual genomes. Therefore, it has been suggested that integrating the two can compensate for each of their shortcomings [19–21]. However, previous studies focused on specific environmental microbes and no efforts have been made to acquire multiple draft genomes of the human microbiota using this hybrid approach, referred to single-cell (sc) metagenomics. Moreover, its advantages over conventional metagenomics binning have not been verified. In this study, we developed a single-cell genomics and metagenomics integration framework (SMAGLinker) to recover HQ genomes of multiple bacterial strains from the microbial community at once. We used microfluidic technology-aided approaches to obtain a large number of SAGs for guided binning [22, 23]. Mock community and human microbiota samples were tested to compare sequence accuracy and number of HQ genomes between conventional metagenomics and sc-metagenomics with SMAGLinker. We also applied sc-metagenomics to acquire strain-resolved genomes and to validate host-plasmid association and the presence of aggregate sequences originating from multiple distinct species in metagenomic bins.

## Results

### Overview of the single-cell genomics and metagenomics integration framework

**Fig. 1:**
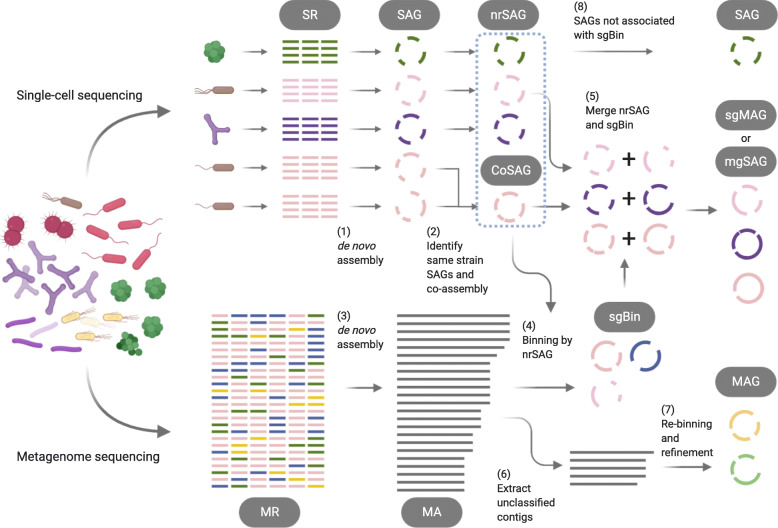
Overview of the single-cell genomics and metagenomics integration framework (SMAGLinker). Single-cell sequencing reads (SRs) and metagenomic sequencing reads (MRs) are obtained from the same microbial community. (1) *De novo* assembly of each SR to a single-cell amplified genome (SAG). (2) SAGs of the same strain are identified into the group and co-assembled into a composite SAG (CoSAG). (3) *De novo* assembly of MRs into metagenome-assembled contigs (MAs). (4) MAs are classified to single-cell genome-guided bin (sgBin) by mapping MA on non-redundant SAG (nrSAG). (5) Paired nrSAGs and sgBins are merged to single-cell genome-guided MAG (sgMAG) or metagenome-guided SAG (mgSAG). (6) Unbinned contigs in MAs are extracted, and subsequently (7), re-binned and refined using conventional metagenome binning and refinement tools. (8) Four types of draft genomes (SAG, sgMAG, mgSAG, and MAG) are finally acquired

For conventional metagenomic phylogenetic classification tools [24, 25] and metagenome binners [10–12], allocating contigs to bins from complex microbial communities in the absence of known microbial genome information as teaching data for classifying closely related species or strains is a challenge. Our single-cell genomics and metagenomics integration framework, called SMAGLinker, uses SAGs, which are also produced from the same sample, as teaching data for metagenome binning (Fig. 1). SAGs of uncultured microbes serve as ideal references for metagenome binning from the community that includes the microbes without reference genomes. These SAGs were obtained using the SAG-gel platform [22, 26], which helps obtain uncontaminated SAGs in a high throughput manner with the aid of a microfluidic droplet format. Multispecies SAGs obtained by assembling single-cell genomes are grouped into individual strains using the ccSAG method [18]. Composite SAGs (CoSAGs) are constructed by re-assembling (co-assembling) single-cell reads (SRs) recognized as identical strains. Based on genome completeness (>50%) and contamination level (<10%), non-redundant SAGs (nrSAGs) are collected for use as binning references. In addition, metagenomic reads (MRs) are obtained from the same sample and are assembled into metagenomic assembled contigs (MAs). The contigs in MAs are mapped to the contigs in nrSAGs to allocate contigs to single cell genome-guided bins (sgBins). Finally, paired nrSAGs and sgBins at the strain level are merged, to plug gaps for each other and extend contig length. The merging of sgBin and nrSAG was performed through guided scaffolding. The more complete genome between sgBin and nrSAG was selected as the primary assembly and the other as the secondary assembly to complement it. To reduce the number of misassemblies and mismatches after merging, contigs larger than 10 kbp were used as secondary assemblies. We used the HaploMerger2 scaffolding tool [27] for merging overlaps >100 kbp with good accuracy. Thus, the integrated sc-metagenome draft genomes are output in two formats: single-cell genome-guided MAG (sgMAG), which is an sgBin-based draft genome complemented with single-cell genome contigs, or metagenome-guided SAG (mgSAG), which is an nrSAG-based draft genome complemented with metagenome contigs.

### Evaluation of single-cell genome and metagenome assemblies

To confirm assembled sequence accuracy in nrSAGs and MAs, single-cell genomic and metagenomic sequencing were performed with the same cell mock community containing 15 bacterial species including *Bacteroides uniformis, Bifidobacterium pseudocatenulatum, Clostridium clostridioforme, Cutibacterium acnes* subsp. *acnes, Escherichia coli* K-12*, Parabacteroides distasonis, Staphylococcus epidermidis, Streptococcus mutans, Acinetobacter radioresistens, Comamonas terrigena, Bacillus subtilis* subsp. *subtilis, Clostridium butyricum, Corynebacterium striatum, Lactobacillus delbrueckii* subsp. *delbrueckii,* and *Pseudomonas putida*. (Additional file 1: Table S1). In total, we obtained 48 SRs and one MR with total read lengths of 3.9 and 2.6 Gb, respectively (Additional file 2: Table S2).

After performing the assembly using SPAdes, 15 nrSAGs, which covered all species in the mock community, were obtained. Average completeness improved from 33.5% to 66.6% from SAG to CoSAG, according to taxonomy identification (Additional file 3: Table S3), with low contamination rates of 0.3% and 0.76%, respectively (Additional file 4: Table S4). For 14 nrSAGs, approximately ≥98.5% of the total length of each was correctly mapped to reference genomes. In Mock-C00006 (*L. delbrueckii*), some contigs (8.5% of the total length) were mapped to other microbial genomes. The original SAGs were obtained from physically isolated single-cells in gel capsules [22]; however free DNA was randomly captured and amplified simultaneously. The unmapped contigs could have been derived from these free DNA fragments. We confirmed that 1008 contigs of 1016 MA contigs were mapped to single reference genomes (Additional file 5: Fig. S1). In addition, there were no 16S rRNA gene sequences for *B. uniformis* and *E. coli* in MA, whereas all nrSAGs remained individual 16S rRNA sequences (Additional file 5: Fig. S2). Overall, both single-cell genomics and metagenomics revealed high sequence accuracy during *de novo* assembly and the presence of sequences sufficiently covers each microbial genome, including highly conserved genes such as the 16S rRNA gene. Thus, we considered the subsequent contig binning step crucial for reconstructing genomes accurately from the metagenomic data set.

### Comparing characteristics of single-cell genome-guided bins with conventional metagenomic bins

**Fig. 2:**
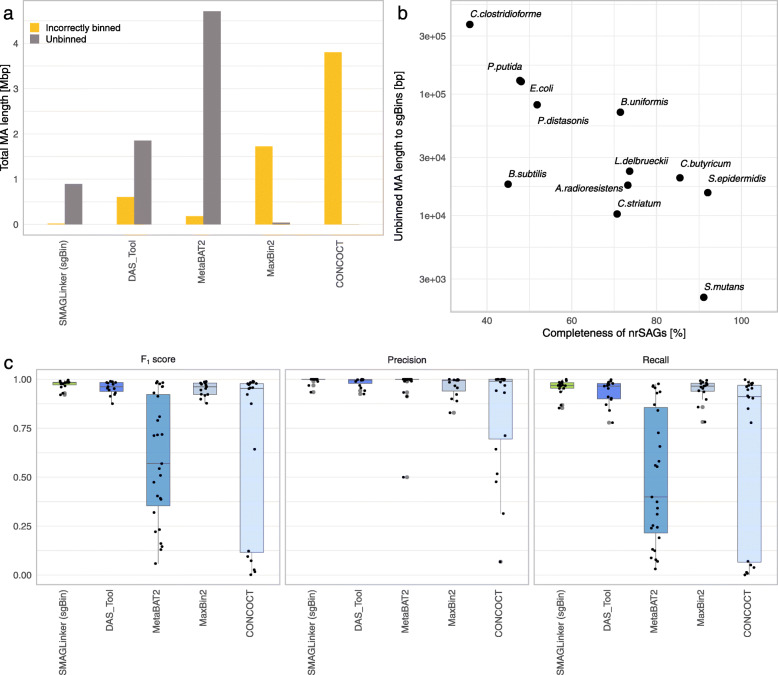
Precision and recall in genome reconstruction with single-cell-guided binning of metagenome assembly. The characteristics of metagenome bins collected using SMAGLinker and conventional 4 metagenome binners (DAS_tool, MetaBAT 2, MAXbin 2, and CONCOCT) were evaluated using the same dataset obtained from a mock microbial community containing 15 bacterial species. (**a**) Total metagenome assembly (MA) lengths of the contigs incorrectly assigned to different bacterial bins, namely “incorrectly binned contig”, and contigs unbinned to any reference genomes, namely “unbinned contig”. (**b**) Correlation between the completeness of non-redundant single-cell amplified genomes (nrSAGs) and total length of unbinned contigs against the target single-cell genome-guided bin (sgBin) in SMAGLinker. *B. pseudocatenulatum, C. acnes, and C. terrigena* had no unbinned contigs. (**c**) The plots of F_1_ scores, precision, and recall of all reported bins in SMAGLinker and four conventional metagenome binners (center line, median; box limits, upper and lower quartiles; whiskers, minimum or maximum values between upper and lower quartiles, which are extended 1.5 times the interquartile region). Individual values are represented as dots

We evaluated the characteristics of bins collected using sc-metagenomics with SMAGLinker and metagenomics-alone approaches with conventional metagenome binners (Fig. 2). CONCOCT [10], MetaBAT 2 [11], and MaxBin 2 [12], were used to construct bins; subsequently, DAS_Tool [13] was used to obtain refined bins. Based on 15 reference genomes (Additional file 1: Table S1), we assessed the taxa of each bin and estimated the total size of contigs incorrectly assigned to different bacterial bins, namely “incorrectly binned contig”, and contigs unbinned to any reference genome, namely “unbinned contig” (Fig. 2a). All contigs were either assigned to one bin or left unassigned according to the binning algorithm; no contig was assigned to more than one bin by any binning tool and SMAGLinker. Because >99% of the contigs were mapped to the reference genome, total MA length of unbinned contigs represents the size of the sequences that should have been incorporated but were missed during binning. SMAGLinker had the smallest incorrectly binned contig at 20 kbp, followed by MetaBAT 2 (181 kbp). CONCOCT had the smallest unbinned contig length at 1kbp. The unbinned contig length for SMAGLinker was 892 kbp. In sc-metagenomics with SMAGLinker, total lengths of unbinned contigs against target sgBins were inversely correlated with the completeness of the corresponding nrSAG (Fig. 2b), suggesting that nrSAG completeness strengthens the adequacy of the contig assignment to target taxa bins. For all tools, incorrectly binned and unbinned contigs tended to have shorter sequence lengths (<10 kbp) (Additional file 5: Fig. S3). Conventional metagenome binners showed high values of either incorrectly binned or unbinned contigs (Fig. 2a), confirming two types of algorithms: one that actively allocates short contigs, while allowing for incorrect allocation (MaxBin 2 and CONCOCT), and the other that allocates contigs carefully to avoid inclusion of incorrect short sequences (DAS_Tool and MetaBAT 2). Furthermore, we found that even long contigs (>100 kb) were incorrectly binned or not assigned to any bin in CONCOCT and MetaBAT 2. In MetaBAT 2, the longest and the second longest contigs derived from *B. subtilis* were not binned, resulting in the total length of unbinned contigs being significantly longer than that derived using other tools. SMAGLinker improved overall binning accuracy because it assigned shorter contigs with higher accuracy than other methods (Additional file 5: Fig. S3).

We calculated F_1_ scores, a harmonic mean of precision and recall, to evaluate the accuracy of bins against true reference genomes (Fig. 2c). Precision depends on a small number of false-positive contigs (incorrectly binned contigs), that is, allocation of contigs from other species in the bin. Although forcing contigs into bins helps improve completeness, it involves the risk of including false-positive contigs, increasing contamination rates. SMAGLinker demonstrated high-precision bins for all 15 reference sequences. High-precision bins (F_1_ score >0.9) for SMAGLinker, DAS_Tool, MetaBAT 2, MaxBin 2, and CONCOCT were 15, 14, 8, 13, and 12, respectively; all metagenome binners, excluding MetaBAT 2, had comparable high-precision values. By contrast, recall value depends on the true completeness of the bacterial genome, ignoring incorrectly binned contigs. SMAGLinker demonstrated the highest F_1_ scores among all reference genomes owing to the highest recall value. In this test, SAG qualities were limited to low-quality (LQ) to medium-quality (MQ), which were not the best conditions to guide binning; however, SMAGLinker had the best binning accuracy. Thus, single-cell guided binning in the sc-metagenomics approach helps in the accurate and efficient allocation of contigs into multispecies bacterial genomes compared to conventional metagenomics-alone approach.

### Integration of SAGs and MAGs to improve the quality of draft genomes

**Fig. 3:**
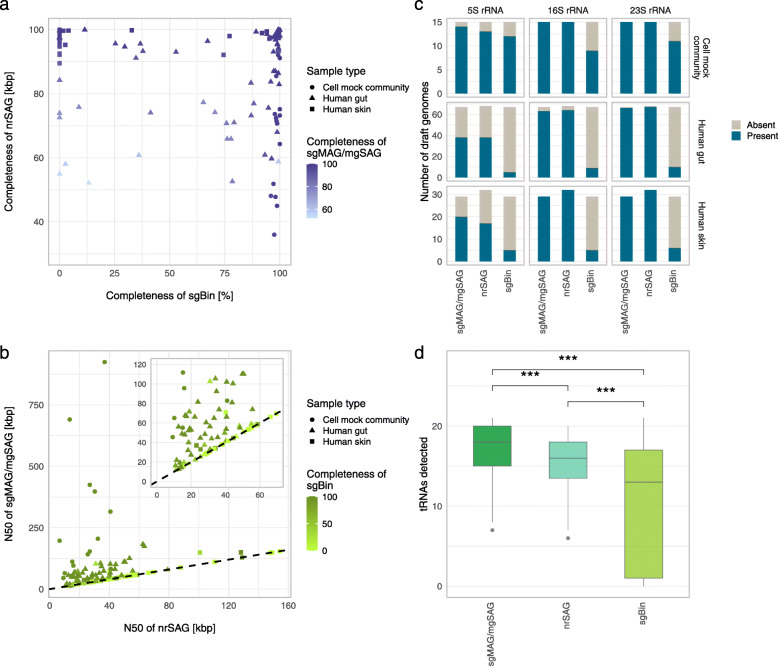
Quality metrics of single-cell genome-guided metagenome-assembled genomes (sgMAGs) and metagenome-guided single-cell amplified genomes (mgSAGs). Data were collected from a mock microbial community containing 15 bacterial species, three human fecal samples, and three human skin swab samples and processed using SMAGLinker. (**a**) Scatter plot of completeness of non-redundant single-cell amplified genomes (nrSAGs) versus single-cell genome guided bins (sgBins) corresponding to medium-quality (MQ) and high-quality (HQ) sgMAGs and mgSAGs. (**b**) Relationship between N50s of nrSAG and sgMAG or mgSAG. Number of rRNA (**c**) and tRNA (**d**) genes in draft genomes produced in the SMAGLinker workflow (center line, median; box limits, upper and lower quartiles; whiskers, minimum or maximum values between upper and lower quartiles, which are extended 1.5 times the interquartile region, Wilcoxon rank sum test ***p < 0.001)

To compensate for the respective incompleteness of SAGs and MAGs, we tested a procedure for constructing draft genomes by integrating paired SAGs and metagenomic bins. The merging of paired nrSAGs and sgBins into sgMAG or mgSAG improved several genome assembly quality metrics, such as completeness and N50, in several microbial communities, including human gut and skin microbiota (Fig. 3a, b). Although the completeness of either nrSAG or sgBin was low (average: 74.5%), that of sgMAG and mgSAG was much improved (average: 93.6%) (Fig. 3a). N50 metrics of most nrSAGs (average: 48.2 kb) improved after merging nrSAG and sgBin (average: 87.7 kb), except in the case of low completeness of sgBins (Fig. 3b). Low completeness of sgBins occurred often, particularly in skin microbiota (average completeness: 23.1%). This may because metagenomic data cannot produce qualified MAs owing to interfering factors, such as human DNA contamination (up to 10% of total MRs) and high within-species diversity in skin microbiota, and the presence of few corresponding contigs at the strain level between SAGs and MAs. Thus, to recover sgBins with high completeness, it is necessary to increase the MA mapping rate by improving its breadth of coverage in assembled contigs and by increasing the SAG repertoire corresponding to MAs. In addition, rRNA and tRNA gene sequences were often compensated from nrSAGs (recovery rate of rRNA: 5S: >53.1%, 16S: >94.1%, and 23S: >98.5% in nrSAGs; and 5S: >7.5%, 16S: >13.4%, and 23S: >14.9% in sgBins) (Fig. 3c, d), thus merging of nrSAGs and sgBin is important for incorporating phylogenetic information of draft genomes.

### Recovery of HQ draft genomes from multiple microbial communities through the sc-metagenomics approach

**Fig. 4:**
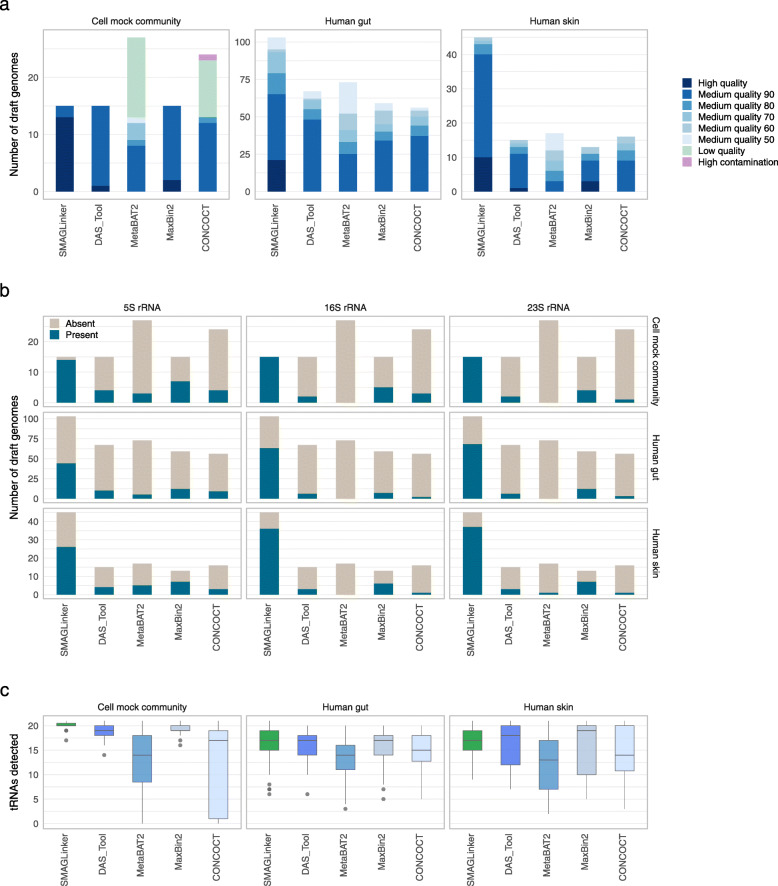
Draft genomes reconstructed from the cell mock community and human microbiota samples. Data were collected from a mock community containing 15 bacterial species, three human fecal samples, and three human skin swab samples. (**a**) Number of reconstructed genomes per method. Human gut and skin data show medium-quality (MQ) and high-quality (HQ) genomes only. Number of rRNA (**b**) and tRNA (**c**) genes in draft genomes produced using SMAGLinker and other tools (center line, median; box limits, upper and lower quartiles; whiskers, minimum or maximum values between upper and lower quartiles, which are extended 1.5 times the interquartile region)

We assessed the quality of all draft genomes according to the Genomic Standards Consortium[9]. From the mock community sample, SMAGLinker, DAS_Tool, and MaxBin 2 constructed MAGs corresponding to 15 reference genomes, whereas MetaBAT 2 and CONCOCT constructed more than 15 MAGs, including several LQ MAGs (Fig. 4a). Thus, the risk of creating unreliable MAGs must also be deliberated when considering the conventional metagenomics-alone approach. SMAGLinker uses nrSAG taxonomy to identify representative species and extract contigs in MAs necessary for binning such that the risk of producing artificial MAGs that cannot be present in actual samples is diminished. Regarding draft genome quality, SMAGLinker produced 13 HQ draft genomes, with better accuracy than other metagenomics-alone approaches (Fig. 4a). For non-chromosomal elements, all plasmid sequences existed in MA; however, these were lost in the plasmid-containing bacterial genomes after metagenomic binning (Additional file 5: Fig. S4). Our sc-metagenomics approach demonstrated constant and higher plasmid coverage (97.2%) than other metagenomics-alone approaches with conventional binners (50.6%–74.5%) in 6 plasmid-harboring bacterial species, including *B. subtilis*, *C. butyricum*, *S. epidermidis*, *A. radioresistens*, *B. uniformis*, and *E. coli*.

To evaluate the performance of SMAGLinker in human gut and skin microbiota, three SR (each 96 SR, 100 Mb/SR) and three MR (each 6 Gb) sets were used to obtain draft genomes with SMAGLinker and other binners. Here, MQ and HQ draft genomes were considered for comparison. The sc-metagenomics approach with SMAGLinker constructed the largest number of genomes, with a total of 103 (21 HQ) and 45 (10 HQ) genomes from the gut and skin, respectively (Fig. 4a and Additional file 6: Table S5). For gut microbiota, no HQ genome was constructed using metagenomics-alone approaches with conventional binners. Although some draft genomes exhibited >90% completeness and <5% contamination using metagenomics-alone approaches, recovery of rRNA and tRNA sequences was a challenge (Fig.4 b,c). The sc-metagenomics approach with SMAGLinker demonstrated consistently high performance in the recovery of rRNA (5S: >42.7%, 16S: >61.2%, and 23S: >66.0%) and tRNA (average: 17.3 ± 2.9) in each microbial sample. SMAGLinker used a large number of sequencing reads by incorporating single-cell genomics and metagenomics; however, trends were unchanged, even when the read number used for other binners was equal to that when SMAGLinker was used (Additional file 5: Fig. S5).

### Coverage of sc-metagenomics-derived draft genomes against bacterial diversity

**Fig. 5:**
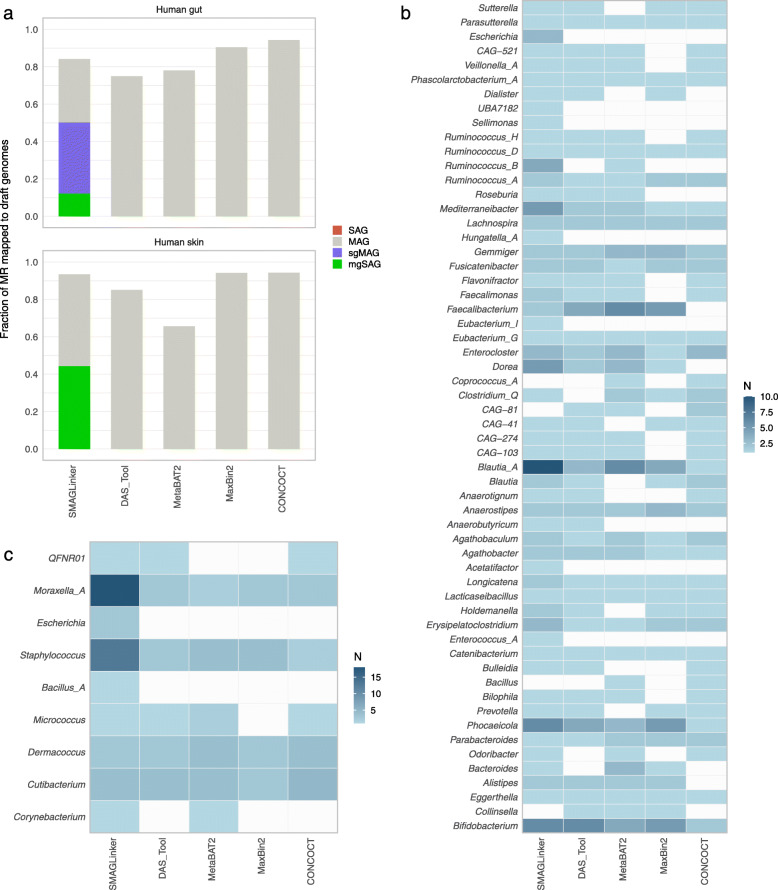
Diversity of microbial draft genomes reconstructed with SMAGLinker. (**a**) Fraction of metagenomic reads mapped on draft genomes constructed with SMAGLinker and four conventional metagenome binners (DAS_tool, MetaBAT 2, MAXbin 2, and CONCOCT). SMAGLinker shows a fraction of metagenomic reads against four types of draft genomes. The number of draft genomes acquired from human (**b**) gut and (**c**) skin are collapsed by genus assigned with GTDB-Tk

To determine the extent to which the constructed genome covered all metagenomic sequence fractions, MRs were mapped to their respective genomes and mapping rates were calculated. For MAGs constructed using MaxBin 2 and CONCOCT, >90% of MRs were mapped (Fig. 5a). These high mapping rates were considered owing to their algorithm trends of unbinned contig reduction (Fig. 2a). The MR mapping rates in SMAGLinker were in the middle of all binners, ranging from 78.9% to 89.5% for gut microbiota and 91.3% to 95.6% for skin microbiota. Regarding bacterial diversity, sc-metagenomics with SMAGLinker detected more bacterial genomes than metagenomics alone approaches with other binners, with 54 and 9 genera in gut and skin microbiota, respectively (Fig. 5b, c). We considered that the sc-metagenomics could cover more metagenomic sequence fraction and obtain diverse microbial genomes by increasing the number of obtained SAGs from the same samples and the number of detected taxa.

### Strain-resolved genome analysis based on sc-metagenomics for revealing intra-species diversity

Genomic classification of closely related species and subspecies from the microbial community is important for discussing intra-species diversity. We assessed the correspondence between MAG and SAG sequences of the same species to evaluate separation accuracy of closely related genomes.

**Fig. 6:**
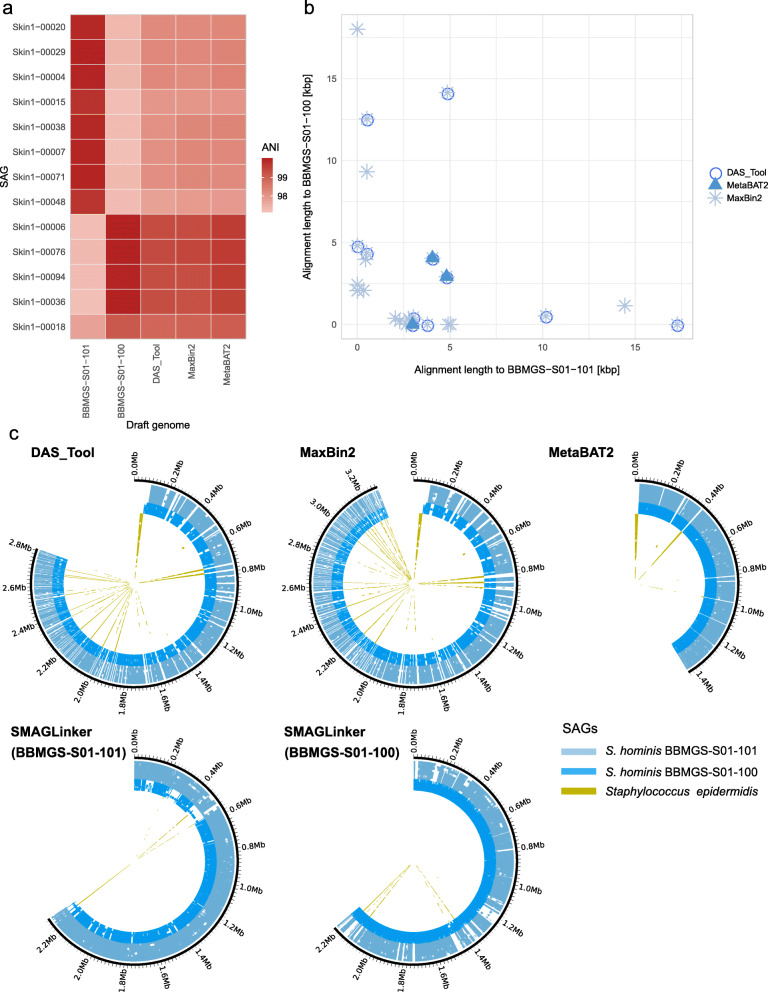
Strain-resolved analysis of skin microbes for host-plasmid linking and detection of interspecies chimeric sequences. (**a**) Mean pairwise genomic similarities between *Staphylococcus hominis* draft genomes obtained with SMAGLinker (*S. hominis* BBMGS-S01-101 and *S. hominis* BBMGS-S01-100) and other binners (DAS_tool, MetaBAT 2, and MAXbin 2). (**b**) The scatter plot shows the length of plasmid contigs assigned to BBMGS-S01-101 and BBMGS-S01-100. Different plot symbols show contigs obtained with different binners. (**c**) Size comparison and interspecies chimeric sequence detection in draft genomes by alignment of *S. hominis* output genomes with BBMGS-S01-101 SAGs and BBMGS-S01-100 SAGs. Outermost black circles show the sizes of draft genomes obtained with each tool; inner circles show the result of mapping individual SAGs, which belong to the same genus of *Staphylococcus*, to the draft genome

In skin microbiota, all metagenome binners output one draft genome of *Staphylococcus hominis*, whereas sc-metagenomics output two draft genomes of *S*. *hominis* (*S. hominis* BBMGS-S01-101 and *S. hominis* BBMGS-S01-100 mgSAGs). We hypothesized that conventional metagenomics-alone approaches had difficulty in binning contigs to two different strains in the same skin microbiota sample. We calculated average nucleotide identity (ANI) of the two strain genomes obtained using SMAGLinker and other metagenome binners against the original SAGs (Fig. 6a) and confirmed that ANI showed >97% identities. We found that although the presence of two strains is evident at the single-cell level, sc-metagenomics could output strain-resolved genomes, and conventional metagenomics produced chimeric MAGs, which demonstrated increased similarity to only one strain (*S. hominis* BBMGS-S01-100). Notably, we found plasmids in MAGs; however, plasmid assignment to mgSAGs indicated that these two strains had specifically different plasmids (Fig. 6b). Thus, our sc-metagenomics framework will aid in strain-resolved binning and plasmid-host allocation to increase, understanding of intra-species diversity and linking mobile gene elements to hosts.

### Validation of aggregate sequences originating from multiple distinct species

SAG can be used as a self-check reference to evaluate the accuracy of conventional MAG binning results, and possibly to remove unsuitable contigs such as aggregate sequences from multiple species. A simple way to detect incorrect sequences in MAG is to map corresponding SAG sequences to MAGs (Fig. 6c). For *S. hominis* obtained from human skin microbiota, we screened SAG sequences that were mapped on MAGs obtained with conventional binners. This result indicated that *S. hominis* MAGs showed different genome sizes with different metagenome binners (1.4 to 3.2 MB) while showing high levels of completeness (83% to 94%), suggesting a substantial lack or excess of genome sequence, and some contaminating sequences from other *Staphylococcus* species (*S. epidermidis*) in all MAGs (55.6 kb–146.8 kb). In particular, the longest contaminated contig (44 kb) in MAGs obtained using DAS_tool and MaxBin 2 showed homology (identity 98.5%) to the pSE2 plasmid of *S. epidermidis* (CP066374). The genome sizes of publicly available *S. hominis* isolate genomes are 2.1–2.3 Mb and are similar to the draft genome obtained with SMAGLinker. BBMGS-S01-101 and BBMGS-S01-100 exhibited some common sequences between *S. hominis* and *S*. *epidermidis*; however, there were no obvious interspecies aggregate sequences. Using SAGs as references, contigs that have been erroneously removed or included by conventional binners can be correctly assigned, suggesting that even uncultured bacterial genomes can be validated at the strain-level.

## Discussion

HQ reference genomes are essential for understanding the phylogeny and function of uncultured microbes in complex microbial ecosystems. In a changing environment, microbes acquire adaptive evolution through repeated genetic mutations and horizontal transfer, etc. [28–31]. To understand the connections between microbial communities and their habitats, recovering genomes from the communities themselves, rather than referring to genomes of closely related bacteria isolated from different environments is preferred.

Despite the cell mock community being a simple sample consisting of 15 different bacteria, the occurrence of incorrectly binned contigs in the conventional MAG suggested the requirement for the careful selection of metagenome binners depending on the presence of conserved genes and the consistency of nucleotide composition. As reported previously [13, 15], a tool that utilizes the bin refinement strategy demonstrated high accuracy, which was in agreement with MAG and the reference genomes. These tools utilize multiple binners to generate various combinations of bins for reference to each other from single or multiple metagenomics data. Alternatively, sc-metagenomics with SMAGLinker generates self-references from the same sample at the single-cell level and guides metagenomic contigs to bins for genome reconstruction. Our sc-metagenomics approach enabled us to obtain the highest quality in draft genomes, both in the mock and human microbiota samples, by assigning metagenomic sequences in correct bins, as well as by filling the gap in highly common sequences, such as rRNA genes, and linking the host with extrachromosomal elements, such as plasmids. The integration of metagenomics and single-cell genomics has been used to improve genome recovery from environmental bacteria. Studies have reported that metagenomic reads can be used to fill in gaps in SAGs [19] and SAGs can be used as scaffolds for MAGs [20]. However, the number of constructed genomes in these studies was limited, and no tool has been developed to obtain multispecies genomes at once, which is mostly due to the lack of technology that provides good quality SAGs as binning guides. In this study, the qualities of SAGs obtained by our SAG-gel technology [22] were sufficiently high to prevent incorrectly binned contigs in supervised contig identification. In addition, merging SAGs with the metagenomic bin aided in the recovery of rRNA and tRNA sequences, which were frequently lacking in the MAGs obtained by conventional binners. This advantage overcomes the incompleteness of phylogenetic information contained in conventional metagenomic bins, suggesting that this technology can be used to move forward from conventional microbial profiling using 16S rRNA gene amplicon sequencing to metabolic function analysis referring to novel genomes.

One of the challenges of sc-metagenomics is the difficulty in obtaining genome sequences beyond the number of SAGs acquired in advance. To obtain genomes from samples of high microbial diversity or genomes of rare microbes, it is necessary to obtain either a large number of SAGs or SAGs of the desired taxa. SMAGLinker can be used by changing the recommended setting values for each parameter according to the quality of SAGs used for analysis (Additional file 5: Supplementary information 1). In this study, we recruited SAGs with >20% completeness to produce CoSAG with >50% completeness. To obtain more draft genomes, the approaches are considered to accumulate massive SAGs with low sequencing efforts to produce nrSAG which covers a broad microbial spectrum, or target single-cell genome sequencing with species enrichment techniques [17, 32, 33]. Another issue with SMAGLinker is that it only allows allocation to a single sgBin per contig for binning using nrSAG as a guide. Under this binning condition, if multiple bacterial strains with extremely similar sequences are present, the assignment of MA contig to sgBin may not be fulfilled in any of the strain genomes. Nonetheless, the implementation of contig assignment to multiple sgBin requires careful consideration owing to the complexity of the computational process and the possibility of producing interspecies aggregate sequences. We recommend using mgSAG, where the completeness of the SAG itself is increased and used as primary data, and the metagenome is used as supplementary information. This procedure allows us to obtain strain-resolved genomes and observe differences among strains, taking advantage of the resolution of SAGs.

The sc-metagenomics approach can control the SAG integration level by adjusting parameters. It is possible to construct representative sequences for each taxonomy rank by setting single copy marker gene homology, ANI, and tetranucleotide frequency, which are parameters used for SAG integration to CoSAG. These SAGs can be utilized as reference genome sequences against which resulting MAGs are checked for harboring interspecies aggregate sequences. Verification of the reliability of MAGs is critical because composite genomes that aggregate sequences from several different populations can provide misleading insights when treated and reported as a single genome. For biological samples that are the source of metagenomic data are properly stored and new single-cell data can be obtained, SMAGLinker can increase the accuracy of acquired data curation and MAG by obtaining new single-cell genomes. In addition, SMAGLinker can subdivide genomes of individual strains, even for species that cannot be divided at the strain level by metagenomic bins. Single-cell based strain-resolved genome analysis will contribute to our understanding of intraspecies diversity and distribution of non-chromosomal elements [30, 34–36].

## Conclusion

In conclusion, sc-metagenomics with SMAGLinker integrates SAG and MAG to reconstruct qualified microbial genomes and control their binning resolution based on the number and classification of SAGs. Because SMAGLinker can provide reliable HQ genomes from various microbial communities, it can be a powerful tool in microbial research that requires reference genome expansion and strain-resolved analysis for understanding microbial association to the host or environment. Thus, SMAGLinker is highly scalable and can be applied to reuse previously acquired metagenomics data and develop single-cell genomics tools.

## Methods

### Experimental design and sample collection

Fresh feces were collected by subjects in 15 mL vials containing 3 mL GuSCN solution (TechnoSuruga Laboratory Co., Ltd,) and stored for 2 d maximum, prior to DNA extraction and single-cell encapsulation in droplets.

Skin bacterial samples were collected and placed in Dulbecco's phosphate-buffered saline (DPBS) by swabbing the surface of facial skin using sterile cotton applicators (Nissui Pharmaceutical Co., Ltd) pre-moistened with DPBS by the participants and were stored at room temperature for 2 d maximum, prior to DNA extraction and single-cell genome amplification.

The mock microbial community (Cell-Mock-001) was obtained from the National Institute of Technology and Evaluation Biological Resource Center, Japan, and contained 15 bacterial species detected in various environments (intestinal, oral, skin, and natural environment).

### Single-cell genome sequencing with SAG-gel

Single-cell genome sequencing was performed with single-cell whole genome amplification (WGA) using the SAG-gel platform, according to our previous reports [22, 26]. Following homogenization of human feces in GuSCN solution (500 μL), the supernatant was recovered by centrifugation at 2000 ×*g* for 30 s, followed by filtration through 35-μm nylon mesh and centrifugation at 8,000 ×*g* for 5 min. The cell pellets were suspended in PBS, washed twice at 8,000 ×*g* for 5 min. Skin swab samples in DPBS were processed in the same manner, except for homogenization.

Prior to single-cell encapsulation, cell suspensions were adjusted to 0.1 cells/droplets in 1.5% agarose in PBS to prevent the encapsulation of multiple cells in a single droplets. Using the droplet generator (On-chip Biotechnologies Co., Ltd.), single microbial cells were encapsulated in droplets and collected in a 1.5-mL tube, which was chilled on ice for 15 min to form the gel matrix. Following solidification, collected droplets were broken with 1H,1H,2H,2H-perfluoro-1-octanol (Sigma-Aldrich) to collect beads. Then, the gel beads were washed with 500 μL acetone (Sigma-Aldrich), and the solution was mixed vigorously and centrifuged. The acetone supernatant was removed, 500 μL isopropanol (Sigma-Aldrich) was added, and the solution was mixed vigorously and centrifuged. The isopropanol supernatant was removed, and the gel beads were washed three times with 500 μL DPBS.

Then, individual cells in beads were lysed by submerging the gel beads in lysis solutions: first, 50 U/μL Ready-Lyse Lysozyme Solution (Epicentre), 2 U/mL Zymolyase (Zymo research), 22 U/mL lysostaphin (MERCK), and 250 U/mL mutanolysin (MERCK) in DPBS at 37 °C overnight; second, 0.5 mg/mL achromopeptidase (FUJIFILM Wako Chemicals) in PBS at 37 °C for 8 h; and third, 1 mg/mL Proteinase K (Promega) with 0.5% SDS in PBS at 40 °C overnight. At each reagent replacement step, the gel beads were washed three times with DPBS and then resuspended in the next solution. Following lysis, gel beads were washed with DPBS five times and the supernatant was removed. The beads were suspended in Buffer D2 and subjected to multiple displacement amplification (MDA) using a REPLI-g Single Cell Kit (QIAGEN). Following WGA at 30 °C for 2 h, gel beads were washed three times with 500 μL DPBS. Thereafter, beads were stained with 1× SYBR Green (Thermo Fisher Scientific) in DPBS. Following the confirmation of DNA amplification by the presence of green fluorescence in the gel, fluorescence-positive beads were sorted into 0.8 μL DPBS in 96-well plates using the FACSMelody cell sorter (BD Bioscience) equipped with a 488-nm excitation laser. Following droplet sorting, 96-well plates were proceeded to the second round of WGA or were stored at −30 °C.

Second-round MDA was performed with the REPLI-g Single Cell Kit. Buffer D2 (0.6 μL) was added to each well and incubation was performed at 65 °C for 10 min. Thereafter, 8.6 μL of MDA mixture was added and incubated at 30 °C for 120 min. The MDA reaction was terminated by heating at 65 °C for 3 min. Following second-round amplification, master library plates of SAGs were prepared. For quality control, aliquots of SAGs were transferred to replica plates for DNA yield quantification using the Qubit dsDNA High Sensitivity Assay Kit (Thermo Fisher Scientific). For sequencing analysis, sequencing SAG libraries were prepared from second-round MDA products using the QIAseq FX DNA Library Kit (QIAGEN). Ligation adaptors were modified to TruSeq™–Compatible Full-length Adapters UDI (Integrated DNA Technologies). Each SAG library was sequenced using the Illumina HiSeq 2 × 150 bp configuration (Macrogen).

### 16S rDNA sequencing

To confirm amplification from single-cell genomes and to identify the taxonomy from the mock community sample, 16S rRNA gene fragments V3–V4 were amplified with 341F and 806R primers (Forward, 5′-TCGTCGGCAGCGTCAGATGTGTATAAGAGACAGCCTACGGGNGGCWGCAG-3′; reverse, 5′-GTCTCGTGGGCTCGGAGATGTGTATAAGAGACAGGACTACHVGGGTATCTAATCC-3′) and sequenced by Sanger sequencing from SAGs obtained by SAG-gel. Following taxonomy identification with BLAST, two to four SAGs corresponding every bacterial species were selected for whole-genome sequencing.

### Metagenome sequencing

Total DNA was extracted from mock samples using International Human Microbiota Standard protocol Q [37]. The DNeasy Power Soil Pro Kit (QIAGEN) was used for total DNA extraction from fecal and skin swab samples. Metagenomic sequencing libraries were constructed from extracted DNA samples with 10-μL (1/5 volume) reactions of the QIAseq FX DNA Library Kit. Each metagenomic sequencing library was sequenced using the Illumina HiSeq 2 × 150 bp configuration (Macrogen).

### Pre-processing and assembly of single-cell genomic and metagenomic sequence reads

SRs and MRs were individually processed for eliminating LQ reads by using fastp 0.20.1 [38] with default options or bbduk.sh 38.79 [39] (options: qtrim=r, trimq=10, minlength=40, maxns=1, minavgquality=15). Human genome contaminations were removed from SRs and MRs by mapping with bbmap.sh 38.79. SRs were assembled *de novo* using SPAdes 3.14.0 (options for SAG: --sc --careful --disable-rr --disable-gzip-output -t 4 -m 32), and contigs <1000 bp were excluded from the subsequent analyses [40]. MRs were assembled into contigs *de novo* using SPAdes 3.14.0 (options: --meta, -t 12, -m 96).

### Grouping same strain SAGs into CoSAG

SAGs with the completeness >10% in the mock community, 20% in the human microbiota sample, and contamination of <10% were selected with CheckM [41]. ANI was calculated for selected SAGs using FastANI 1.3 [42]. The homology of common single-copy marker genes obtained using CheckM v1.1.2 taxonomy workflow (option: -nt --tab_table -t 16 domain Bacteria) was calculated by blastn 2.9.0+ with the default option. SAGs with ANI >95%, single-copy marker gene homology >99%, and tetra-nucleotide frequencies correlation >90% were identified in the same strain group. SRs from one SAG were mapped to other SAGs in the same group using MINIMAP2 2.17 (options: -ax sr) [43]. According to the ccSAG procedure [18], potential chimeras that partially aligned were split into aligned and unaligned fragments. The short fragments (<20 bp) were discarded. Clean and chimera-removed reads were obtained using cycles of cross-reference mapping and chimera splitting for each sample in the same group. Quality controlled reads from the same group were co-assembled *de novo* as CoSAG using SPAdes (options: --sc --careful --disable-rr --disable-gzip-output -t 4 -m 32).

### SAG-guided binning of metagenome contigs

The MAs were individually mapped against the strain-specific nrSAG contig using BWA 0.7.17 with the default option [44]. MA contigs with >99% identity (>200bp) to nrSAG contigs were extracted to construct sgBins.

### Merging of nrSAG and sgBin

CheckM was performed to measure the completeness of the two sets of assemblies, nrSAG and sgBin, and the assembly with higher completeness was defined as the primary and that with lower completeness as the secondary. Secondary assemblies were processed using SeqKit [45], and contigs <10000 bp were removed. Primary and secondary assemblies were merged using HaploMerger2_20180603 [27] to create sgMAGs or mgSAGs. Thereafter, MAGs were reconstructed by using the DAS-tool from MAs that were unclassified as sgBin.

### Conventional MAG binning

For comparison of MAG quality, multiple binnings of metagenomic contigs were conducted using conventional binners including CONCOCT 1.0.0 [10], MaxBin 2 v2.2.6 [12], and MetaBAT 2 v2.12.1 [11] with default options. To refine binning results, DAS_Tool 1.1.2 [13] was used with default options.

### Gene prediction, taxonomy identification, and plasmid detection

CDS, rRNAs, and tRNAs were extracted from all SAGs or MAGs through Prokka 1.14.6 [46] (option: --rawproduct --mincontiglen 200). Then, 16S and 23S rRNA genes with lengths ≥700 and 1100 bp, respectively, were detected. Taxonomy identification was performed using GTDB-Tk 1.3.0 [47] with the default option, using the Release95 database. PlasClass [48] was used for detecting plasmids.

### Quality assessment of draft genomes from the mock community

For the mock community sample analysis, ANIs of each draft genome (sgMAG and mgSAG) for the closest reference genome were calculated with FastANI 1.3. The closest taxa with ≥ 99.5% ANI was assigned to each draft genome. The quality of all obtained SAGs and MAGs were evaluated using QUAST v.5.0.2 (default option) [49], CheckM v1.1.2 lineage workflow (option: --nt --tab_table -t 16), and identification of 5S, 16S, and 23S rRNA. To assess the accuracy of draft genomes procured from mock community samples, draft genomes were individually mapped to the corresponding taxa reference genome using MINIMAP2 2.17 with default options. The mapping results were converted to the pileup textual format using SAMtools 1.9 [50], and genomic coverage (L) for the reference genome was calculated using the following equation.

$${L}_i= length\left({A}_i\cap {G}_g\right), where\;g=\mathit{\arg}\ {\mathit{\max}}_{j\in G}\left\{ ANI\left({A}_{i,}{G}_j\right)\right\}$$

where A_i_ represents the i^th^ draft genome. G and G_j_ represent the set of reference genomes and the j^th^ reference genome of the set, respectively. G_g_ represents the corresponding reference genome against A_i_. When the reference genome is G_g_ and the draft genome is A_i_, precision (P), recall (R), and F value (F_1_ score) of the reference genome were calculated using the following equations.

$${P}_i=\frac{L_i}{length\left({A}_i\right)}$$

$${R}_i=\frac{L_i}{length\left({G}_g\right)}$$

$${F}_1{score}_i=2\frac{P_i{R}_i}{P_i+{R}_i}$$

## Supplementary Information

**Additional file 1: Table S1** Cell mock community reference genome.

**Additional file 2: Table S2** Sequence reads obtained from single-cell amplified genomes (SAGs) and metagenomes.

**Additional file 3: Table S3** Single-cell amplified genome (SAG) to composite SAG.

**Additional file 4: Table S4** Assembly quality of composite single-cell amplified genomes (CoSAGs) of cell mock community.

**Additional file 5: Fig. S1** Distribution of coverage and length of contigs mapped on bacterial genomes contained in a cell mock community. **Fig. S2** Number of rRNA genes and tRNA genes in metagenome-assembled contigs (MAs) from 15 bacterial species of a microbial community before binning. **Fig. S3** Histograms of length of contigs binned by SMAGLinker and other metagenome binning tools from 15 bacteria of a microbial community. **Fig. S4** The performance of metagenomic assembly and binning in the recovery of plasmid sequences. **Fig. S5** Draft genomes reconstructed from human microbiota samples with SMAGLinker and other binners with doubled metagenomic data. **Supplementary information 1.** SMAGLinker setting parameters.

**Additional file 6: Table S5** High-quality (HQ) and medium-quality (MQ) draft genomes (SAG, mgSAG, and sgMAG) constructed using SMAGLinker.

## Data Availability

SMAGLinker is available from https://github.com/kojiari/smaglinker. Sequencing data have been deposited in the NCBI database under BioProject PRJNA692334 (see Additional file 6: Table S5 for details).

## Abbreviations

ANI: Average nucleotide identity
CoSAG: Composite SAG
MAG: Metagenome-assembled genome
MA: Metagenomic assembled contig
mgSAG: Metagenome-guided SAG
MR: Metagenomic read
MDA: Multiple displacement amplification
nrSAG: Non-redundant SAG
sc: Single-cell
SAG: Single-cell amplified genome
sgBin: Single-cell genome-guided bin
sgMAG: Single-cell genome-guided MAG
SR: Single-cell read
