# Supplementary material for: Recovery of strain-resolved genomes from human microbiome through an integration framework of single-cell genomics and metagenomics
Source: Microbiome. 2021 Oct 12;9:202. doi: 10.1186/s40168-021-01152-4 (PMC8507239; doi:10.1186/s40168-021-01152-4)
Supplement: Supplementary file 6 — Additional file 5: Fig. S1 Distribution of coverage and length of contigs mapped on bacterial genomes contained in a cell mock community. Fig. S2 Number of rRNA genes and tRNA genes in metagenome-assembled contigs (MAs) from 15 bacterial species of a microbial community before binning. Fig. S3 Histograms of length of contigs binned by SMAGLinker and other metagenome binning tools from 15 bacteria of a microbial community. Fig. S4 The performance of metagenomic assembly and binning in the recovery of plasmid sequences. Fig. S5 Draft genomes reconstructed from human microbiota samples with SMAGLinker and other binners with doubled metagenomic data. Supplementary information 1. SMAGLinker setting parameters. [file 40168_2021_1152_MOESM5_ESM.docx]

**Fig. S1 Distribution of coverage and length of contigs mapped on bacterial genomes contained in a cell mock community.** Regarding contig assignment, 91.1% of the total contigs were fully mapped to the single reference genome with ≥99% homology, while the contigs with the coverage of ≥200% were mapped to the repeating sequence part of the reference genomes. The majority of them were <5k bases in length.

**Fig. S2 Number of rRNA genes and tRNA genes in metagenome-assembled contigs (MAs) from 15 bacterial species of a microbial community before binning.**

**Fig. S3 Histograms of length of contigs binned by SMAGLinker and other metagenome binning tools from 15 bacteria of a microbial community.**

**Fig. S4 The performance of metagenomic assembly and binning in the recovery of plasmid sequences.** Each bar shows the coverage of plasmid estimated from sequences in metagenome assembled contigs (MAs) and bins obtained with SMAGLinker and the other four binners. All data were collected from six plasmid-containing bacteria in a cell mock community.

**Fig. S5 Draft genomes reconstructed from human microbiota samples by SMAGLinker and other binners with doubled metagenomic data.** All data were collected from three human fecal samples and three human skin swab samples. Single-cell genomic sequence reads (8.7 Gb) and metagenomic sequence reads (6.0 Gb) were used in SMAGLinker. Doubled metagenomic sequence reads (approximately 14.9 Gb) were used in the other four binners.

**Supplementary Information 1**

SMAGLinker setting parameters:

Quality of SAG to be adopted as nrSAG: completeness >50%, contamination <10%.

- If many SAGs with low completeness are to be used, lowering the completeness threshold can improve the number of SAG responses to metagenome assembly.

- By contrast, when loosening the setting value, we need to pay attention to the accuracy of binning using incomplete SAGs as guides.

Contig length of SAG: >1000 bp

- By lowering the setting value and incorporating shorter contigs, completeness is improved. By contrast, contamination tends to increase. Adopting a contig longer than the set value increases the reliability of the sequence but may lead to a decrease of contigs corresponding to metagenome assembly.

Quality of SAGs to be subjected to ccSAG processing: completeness > 20%, contamination < 10%.

- Depending on the quality of the SAGs to be used, we can change the setting value; however, if the setting parameters are inadequately low, it may not be possible to integrate the SAGs properly.

- It is recommended to ensure a sufficient number of SAGs to cover the whole genome envisioned by the entire SAGs to be integrated.

Strain identification for constructing CoSAG in ccSAG: ANI > 95%, homology > 99%, TNF correlation > 90%.

-Default parameters are set to cluster genomes at the strain level.

-The lower the parameter's value, the higher the clustering in the taxonomy rank, and the more likely it is to construct a population consensus draft genome.

Condition for mapping MA on nrSAG contigs: identity >99%, >200 bp

- If the condition is strictly set, the integration of SAG and MAG will be less likely to occur, and the result will be closer to the result of the independent analysis of SAG and MAG.

Contig length in secondary assemblies for merging nrSAG and sgBin: >10 kbp

- When the length condition of the secondary contig is long, the total size and completeness rate of the merged genome tends to decrease, and the misassembly and contamination rate tends to decrease; when the length condition of the secondary contig is short, an opposite trend is observed.

- A setting of 2–10 kbp is recommended.

**Tables**

Table S1 Cell mock community reference genome

Table S2 Sequence reads obtained from single-cell amplified genomes (SAGs) and metagenome

Table S3 Single-cell amplified genome (SAG) to composite SAG (CoSAG)

Table S4 Assembly quality of composite single-cell amplified genomes (CoSAGs) of cell mock community

Table S5 High-quality (HQ) and medium-quality (MQ) draft genomes constructed using SMAGLinker
